# Involvement of Receptor Activator of Nuclear Factor-κB Ligand (RANKL)-induced Incomplete Cytokinesis in the Polyploidization of Osteoclasts*[Fn FN2]

**DOI:** 10.1074/jbc.M115.677427

**Published:** 2015-12-15

**Authors:** Noriko Takegahara, Hyunsoo Kim, Hiroki Mizuno, Asako Sakaue-Sawano, Atsushi Miyawaki, Michio Tomura, Osami Kanagawa, Masaru Ishii, Yongwon Choi

**Affiliations:** From the ‡ Next Generation Optical Immune-imaging, WPI-Immunology Frontier Research Center, Osaka University, Suita, Osaka 565-0871, Japan,; the § Department of Pathology and Laboratory Medicine, University of Pennsylvania Perelman School of Medicine, Philadelphia, Pennsylvania 19104,; the ¶ Department of Immunology and Cell Biology, Graduate School of Medicine and Frontier Biosciences, WPI-Immunology Frontier Research Center, Osaka University, 2-2 Yamada-oka, Suita, Osaka 565-0871, Japan,; the ‖ CREST, Japan Science and Technology Agency, 5 Sanban-cho, Chiyoda-ku, Tokyo 102-0075, Japan,; the ** Laboratory for Cell Function and Dynamics, Advanced Technology Development Group, Brain Science Institute, RIKEN, Wako-city, Saitama 351-0198, Japan,; the ‡‡ Laboratory for Autoimmune Regulation, Research Center for Allergy and Immunology, RIKEN, Yokohama City, Kanagawa 230-0045, Japan,; the §§ Laboratory of Immunology, Faculty of Pharmacy, Osaka-Ohtani University, 3-11-1 Nishikiorikita, Tondabayashi-city, Osaka 584-8540, Japan, and; the ¶¶ Department of Molecular Preventive Medicine, Graduate School of Medicine, University of Tokyo, 7-3-1 Hongo, Bunkyo-ku, Tokyo 113-033, Japan

**Keywords:** cell biology, cell division, cell proliferation, flow cytometry, imaging, osteoclast, incomplete cytokinesis, polyploidy

## Abstract

Osteoclasts are specialized polyploid cells that resorb bone. Upon stimulation with receptor activator of nuclear factor-κB ligand (RANKL), myeloid precursors commit to becoming polyploid, largely via cell fusion. Polyploidization of osteoclasts is necessary for their bone-resorbing activity, but the mechanisms by which polyploidization is controlled remain to be determined. Here, we demonstrated that in addition to cell fusion, incomplete cytokinesis also plays a role in osteoclast polyploidization. In *in vitro* cultured osteoclasts derived from mice expressing the fluorescent ubiquitin-based cell cycle indicator (Fucci), RANKL induced polyploidy by incomplete cytokinesis as well as cell fusion. Polyploid cells generated by incomplete cytokinesis had the potential to subsequently undergo cell fusion. Nuclear polyploidy was also observed in osteoclasts *in vivo*, suggesting the involvement of incomplete cytokinesis in physiological polyploidization. Furthermore, RANKL-induced incomplete cytokinesis was reduced by inhibition of Akt, resulting in impaired multinucleated osteoclast formation. Taken together, these results reveal that RANKL-induced incomplete cytokinesis contributes to polyploidization of osteoclasts via Akt activation.

## Introduction

Polyploidy, in which a cell has more than the diploid complement of chromosomes, is a widespread physiological phenomenon observed especially in plants, fungi, and insects (1). Although it is less common in mammals, polyploidization occurs in selected tissues, including the placenta, liver, heart, skeletal muscle, and bone marrow during normal development and aging (2). During developmental programs, cells obtain additional sets of chromosomes by various mechanisms, including endocycles, endomitosis, incomplete cytokinesis, and cell fusion. In endocycles and endomitosis, the cell undergoes successive rounds of DNA replication without intervening mitosis or karyokinesis (abort mitosis during metaphase). These cycles do not produce two nuclei in the cell. The best-studied examples of endocycles and endomitosis are trophoblast giant cells and megakaryocytes, respectively (3–6). In incomplete cytokinesis, the cell undergoes karyokinesis but skips cytokinesis, resulting in a cell with two nuclei; this process has been implicated in the normal development of hepatocytes and cardiomyocytes (7, 8). Cell fusion, which involves merging of the plasma membrane and cytoplasmic mixing, is observed during development of skeletal muscle cells and osteoclasts (9, 10). Endocycles, endomitosis, and incomplete cytokinesis are directly associated with the proliferative state of the cell. By contrast, cell fusion is entirely independent of cell proliferation (10).

Polyploidy is a hallmark of mature osteoclasts, which are specialized multinucleated giant cells that resorb bone (11). These cells are hematopoietic in origin and are derived from myeloid precursors that also give rise to macrophages and dendritic cells. When myeloid precursors receive signals mediated by the osteoclast differentiation factor RANKL,^3^ which is mainly produced by osteoblasts, they commit to becoming pre-osteoclasts and ultimately differentiate into multinucleated osteoclasts (12). The importance of polyploidization in osteoclast formation is demonstrated by the impaired bone-resorbing activity of osteoclasts that cannot achieve polyploidy (13).

Although generation of polyploid osteoclasts is thought to occur due to cell fusion, independently of cell proliferation (14), some researchers have pointed out a relationship between cell proliferation and osteoclast differentiation. For example, in osteoclast progenitors, progression and subsequent withdrawal from the cell cycle are required for differentiation into osteoclasts (15–17). In addition, stimulation with RANKL also triggers a signaling pathway that is essential for cell cycle progression (18). These reports prompted us to investigate whether cell cycle progression has an impact on polyploidization during osteoclastogenesis and, if so, how and to what extent the cell cycle regulates the polyploidization of osteoclasts. The fluorescent ubiquitination-based cell cycle indicator (Fucci) is a powerful tool for studying coordination of the cell cycle with other developmental processes (19–22). The Fucci probe was generated by fusing red fluorescent protein and green fluorescent protein to human Cdt1 and human geminin, respectively. These two chimeric proteins accumulate reciprocally in the nuclei during the cell cycle, labeling the nuclei of cells in G_0_/G_1_ phase red and those in S/G_2_/M phase green. Thus, these proteins function as effective G_0_/G_1_ and S/G_2_/M markers.

Here, using monocytes derived from Fucci transgenic mice, we show that RANKL-induced polyploidization occurs not only by cell fusion but also by incomplete cytokinesis. In these cells, RANKL stimulation transiently increased basal proliferation and induced incomplete cytokinesis as well as cell fusion. Also, cells that underwent incomplete cytokinesis had the potential to undergo cell fusion. In addition, fluorescence *in situ* hybridization revealed that some of osteoclasts exhibited nuclear polyploidy (*i.e.* they contained nuclei with more than the diploid complement of chromosomes (>2N)) *in vivo*, suggesting that cells that undergo incomplete cytokinesis are involved in physiological polyploidization of osteoclasts. Furthermore, RANKL stimulation induced phosphorylation of Akt, which is required for efficient polyploidization by either incomplete cytokinesis or cell fusion. Collectively, our findings reveal an unexpected pattern of cell division and fusion during the generation of polyploid osteoclasts.

## Experimental Procedures

### 

#### 

##### Mice

Fucci transgenic mouse lines FucciS/G2/M-#474 and FucciG1-#639 were obtained from the RIKEN BioResource Center. These lines were cross-bred to obtain Fucci double-transgenic mice. The generation of TRAP promoter-tdTomato transgenic mice and V-type H^+^-ATPase a3 subunit-GFP fusion protein expressing mice (a3-GFP) were described previously (23). All animal work was performed under veterinary supervision in an accredited facility using protocols approved by the Animal Care and Use Committee of the University of Pennsylvania and Osaka University.

##### In Vitro Osteoclast and Multinucleated Giant Cell Differentiation

Bone marrow-derived macrophages (BMMs) from wild type (WT) or Fucci double-transgenic (dTg) mice were obtained from cultures of bone marrow collected from 6- to 8-week-old male tibias and femurs, as described previously. In brief, bone marrow progenitor cells were cultured with M-CSF (60 ng/ml) in α-minimal essential medium containing 10% FCS. After 3–4 days, cells were gathered as BMMs. For osteoclast differentiation, BMMs were cultured for 3 days in α-minimal essential medium containing M-CSF (60 ng/ml) and RANKL (150 ng/ml). After culture for 3 days, the cells were fixed with 3.7% formaldehyde in PBS for 10 min and then stained for TRAP using the acid phosphatase, leukocyte (TRAP) kit (Sigma). TRAP-positive multinucleated cells containing three or more nuclei were counted. For differentiation of multinucleated giant cells (MGCs), BMMs derived from wild type mice were cultured in the presence of IL-3 (100 ng/ml) (R&D Systems) and IL-4 (100 ng/ml) (PeproTech) for 2 days. MGCs were stained with May-Grunewald Giemsa stain (Life Technologies, Inc.). For inhibitor assays, BMMs were cultured with M-CSF (60 ng/ml) and RANKL (150 ng/ml) or with IL-4 (100 ng/ml) plus IL-3 (100 ng/ml) in the presence of Y27632 (10 μm) (Wako), Akt inhibitor VIII (5 μm) (Sigma), SN50 (50 μg/ml) (BioVision), LY294003 (5 μm) (Wako), aphidicolin (500 nm) (Sigma), or DMSO for 72 h.

##### BrdU Incorporation Assay

Wild Type-BMMs were cultured with M-CSF (60 ng/ml) in the presence or absence of RANKL (150 ng/ml) or cultured with IL-4 (10 ng/ml) plus IL-3 (100 ng/ml) for the indicated amount of time (in hours). BrdU (10 μm) were added to the culture for the last 6 h. After culture, cells were washed with PBS and detached using enzyme-free cell dissociation buffer (Millipore) at 37 °C for 5 min. After generation of single-cell suspensions, cells were counted and suspended at 1 × 10^6^/ml in PBS in polystyrene tubes. The cells were stained with LIVE/DEAD Aqua (Life Technologies, Inc.) for 30 min on ice to exclude dead cells. Cells were washed with PBS with 2% FCS and fixed with BrdU fixation buffer (eBioscience) for 1 h on ice followed by DNase treatment. Cells were stained with FITC-labeled anti-BrdU antibody (clone Bu20a, eBioscience) for 20 min, and after washing, cells were further stained with 7-AAD. Finally, the cells were analyzed using a FACSCanto II (BD Biosciences). Numbers indicate the percentages of S phase cells. Results are representative of three independent experiments.

##### Ploidy Analysis

dTg-BMMs stimulated with RANKL for the indicated times in the presence of M-CSF were washed with PBS and detached using enzyme-free cell dissociation buffer (Millipore) at 37 °C for 5 min. After generation of single-cell suspensions, cells were counted and suspended at 1 × 10^6^/ml in phenol red-free α-minimal essential medium containing 2% FCS and 2 mm EDTA in polystyrene tubes. Then the cells were stained with 5 μm Vybrant DyeCycle Violet (Life Technologies, Inc.) at 37 °C for 60 min. Finally, the cells were stained with TO-PRO-3 (Life Technologies, Inc.) to exclude dead cells and analyzed using an LSR (BD Biosciences). Excitation laser lines and emission filters were as follows: Vybrant DyeCycle Violet: excitation, 405-nm laser line, and emission, 450/50 BP; mAG: excitation, 488-nm laser line, and emission, 515/20 BP;mKO2: excitation, 532-nm laser line, and emission, 585/42 BP; TO-PRO-3: excitation, 640-nm laser line, and emission, 660/20 BP. Data were analyzed using the FlowJo software (Tree Star).

##### Time-lapse Microscopy and Analysis

dTg-BMMs cultured in a glass-bottom dish were stimulated with RANKL (150 ng/ml) in the presence of M-CSF (60 ng/ml) or stimulated with IL-4 (100 ng/ml) and IL-3 (100 ng/ml) for the indicated times, and then subjected to time lapse imaging. Cell-tracking experiments were performed using a Deltavision microscope (Applied Precision) from an Olympus IX70 microscope equipped with a ×20 or ×40 objective, 300-watt xenon lamp, and a Photometrics CoolSNAP HQ 12-bit monochrome-cooled CCD camera. A series of images was collected every 5 min at 37 °C. To acquire large square fields of view, multipoint time-lapse imaging was performed. Image acquisition and processing were performed using the Deltavision SoftWorx software (Applied Precision), and image analysis was performed using the Fiji software (National Institutes of Health). Cells were manually tracked. Mononucleated cell fusion was counted as “cell fusion.” Mononucleated or binucleated cells that went through cell fusion following incomplete cytokinesis, or daughter cells of a binucleated cell that underwent cell fusion, were counted as “cell fusion involving incomplete cytokinesis.” Doubling times of cytokinesis/incomplete cytokinesis were assessed using a Nikon BioStation IMQ imaging system, and image analysis was performed using the Imaris software (Bitplane). The time from the end of cytokinesis/incomplete cytokinesis to the next end of cytokinesis/incomplete cytokinesis was measured as the doubling time. Final graphs were generated using GraphPad Prism (GraphPad Software).

##### Flow Cytometry

dTg-BMMs cultured with M-CSF (60 ng/ml) in the presence or absence of RANKL (150 ng/ml) for 48 h were washed with PBS and detached using enzyme-free cell dissociation buffer (Millipore) at 37 °C for 5 min. After generation of single-cell suspensions, cells were counted and suspended at 1 × 10^6^/ml in PBS in polystyrene tubes. Cells were stained with 1 μm LIVE/DEAD NearIR (Life Technologies, Inc.) on ice for 30 min to exclude dead cells, washed with PBS with 2% FCS, and stained with allophycocyanin-labeled antibodies against integrin β3 or F4/80 (integrin β3, clone 2C9.G3; F4/80, clone MB8 (eBioscience)) on ice for 30 min. After washing, cells were further stained with 5 μm Vybrant DyeCycle Violet (Life Technologies, Inc.) at 37 °C for 60 min. A Fucci transgenic mouse line FucciG1-#610 obtained from the RIKEN BioResource Center and a3-GFP mice line were cross-bred to obtain Fucci-mKO2 and a3-GFP expressing mice (Fucci-mKO2/a3-GFP). BMMs were prepared from Fucci-mKO2/a3-GFP mice and cultured with M-CSF (60 ng/ml) in the presence or absence of RANKL (150 ng/ml) for the indicated times. After generation of single-cell suspensions, cells were stained with 5 μm Vybrant DyeCycle Violet (Life Technologies, Inc.) at 37 °C for 60 min. Finally, the cells were stained with TO-PRO-3 (Life Technologies, Inc.) to exclude dead cells. Peripheral blood cells or bone marrow cells of Fucci-mAG transgenic mice were stained with allophycocyanin-labeled antibodies against Ly6C (clone HK1.4 (eBioscience)) on ice for 30 min. After washing, cells were further stained with 5 μm Vybrant DyeCycle Violet (Life Technologies, Inc.) at 37 °C for 60 min. Finally, the cells were stained with 7-AAD (eBioscience) to exclude dead cells. Cells were analyzed using LSR (BD Biosciences), and data were analyzed using the FlowJo software (Tree Star).

##### Gelatin Degradation Assay

Fluorescein isothiocyanate (FITC) gelatin-coated glass bottom dishes were prepared as described previously (24). dTg-BMMs were cultured on the FITC gelatin-coated dishes for 24 h with M-CSF (60 ng/ml) and RANKL (150 ng/ml) and then subjected to time-lapse imaging using a confocal A1 microscope system (Nikon). A series of images was collected every 30 min at 37 °C. Image analysis was performed using NIS-elements software (Nikon).

##### Fluorescence in Situ Hybridization

TRAP promoter-tdTomato transgenic mice were perfused with 4% paraformaldehyde plus sucrose for fixation, and bone tissues were further fixed with 4% paraformaldehyde plus sucrose for 3 h at 4 °C. Next, the bones were incubated with 10% EDTA for 2 weeks for decalcification and embedded in O.C.T. compound (Tissue-Tek). Sections 5 μm thick were prepared using Kawamoto's film method and treated with pepsin, and then FISH probe mixture was added. The sections were denatured at 90 °C for 10 min and then kept overnight at 37 °C for hybridization. After hybridization, the sections were washed with 5% formamide, 2× SSC for 20 min at 37 °C, and then 1× SSC for 10 min at room temperature. After washing, the sections were stained with DAPI, and images were acquired using the Leica CW-4000 system (Leica) with a ×100 objective.

##### Western Blotting

After treatment of cells with the indicated cytokines or inhibitors for the indicated times, cells were washed twice with ice-cold PBS and scraped from the plastic plate with a cell lifter (Costar), and then whole-cell lysates were isolated in RIPA buffer (1% Nonidet P-40, 0.5% sodium deoxycholate, 0.1% SDS, 25 mm Tris-HCl, pH 7.6, 150 mm NaCl) supplemented with protease inhibitor mixture and PhosSTOP (Roche Applied Science). Equivalent amounts of protein (20–40 μg) were subjected to 10% SDS-PAGE, and immunoblotting was performed using antibodies specific for phospho-Akt (Ser-473), Akt, phospho-p38 MAPK (Thr-180/Tyr-182), p38, phospho-p44/42 MAPK (ERK1/2) (Thr-202/Tyr-204), ERK1/2, JNK (Cell Signaling), and phospho-JNK (Thr-183/Tyr-185) (BD Biosciences). For RhoA activation assay, cell lysates were incubated with Rhotekin-RBD protein beads and blotted with anti-RhoA mAb (Cytoskeleton).

##### Statistical Analysis

Data were analyzed using one-way analysis of variance or unpaired *t* test with Welch's correction and are presented as means ± S.D. A *p* value < 0.05 was considered significant.

## Results

### 

#### 

##### RANKL Stimulation Increases Basal Proliferation of BMMs

**FIGURE 1.**
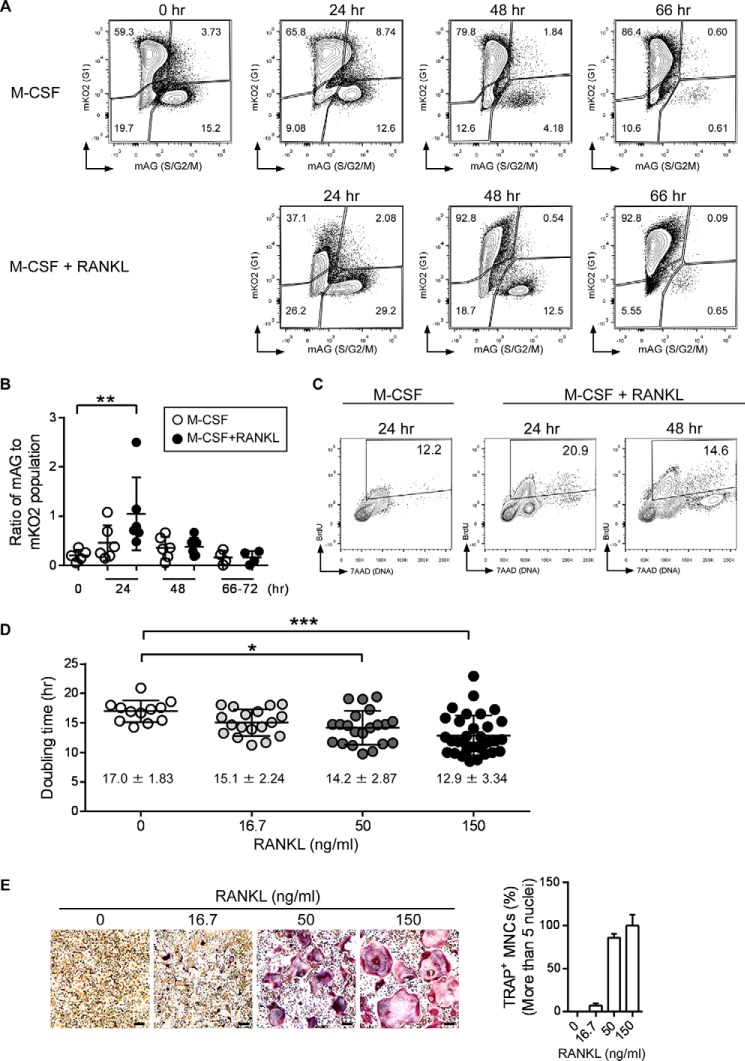
**RANKL stimulation increases basal cell proliferation.** *A,* flow cytometry analysis of dTg-BMMs during osteoclast differentiation. dTg-BMMs were cultured with M-CSF (60 ng/ml) in the presence or absence of RANKL (150 ng/ml) for the indicated times. Cells were harvested at the indicated times, and cells positive for *red* (mKO2) or *green* (mAG) fluorescence were detected by flow cytometry. Results are representative of three to five independent experiments. *B,* ratios of *red* (mKO2) fluorescence-positive cells to *green* (mAG) fluorescence-positive cells. Each *circle* represents the result of an independent flow cytometry experiment. *Bars* indicate means ± S.D. *C,* BrdU incorporation assay. WT-BMMs were cultured with M-CSF (60 ng/ml) in the presence or absence of RANKL (150 ng/ml) for the indicated amount of time (in hours). BrdU (10 μm) was added for the last 6 h. Incorporated BrdU was stained with FITC-labeled anti-BrdU antibody. DNA was stained with 7-AAD and analyzed by flow cytometry. *Numbers* indicate the percentages of S phase cells. Results are representative of three independent experiments. *D,* doubling time of dTg-BMMs during osteoclast differentiation. dTg-BMMs were cultured with M-CSF (60 ng/ml) in the presence the indicated dose of RANKL (ng/ml) for 48 h. Each *circle* represents the result of a cell. *Bars* indicate means ± S.D. *, *p* < 0.05; ***, *p* < 0.001. *E, in vitro* osteoclast differentiation. dTg-BMMs were cultured with M-CSF (60 ng/ml) in the presence of the indicated dose of RANKL (ng/ml) for 96 h. Percentages of multinucleated cells containing more than five nuclei are shown. *Scale bars,* 100 μm.

To determine the impact of RANKL stimulation on the cell cycle during osteoclast development, we first examined the proportions of cells in the G_1_ and S/G_2_/M phases during RANKL-induced osteoclast differentiation. Fucci double-transgenic mouse-derived bone marrow monocytes (dTg-BMMs) were stimulated with or without RANKL in the presence of M-CSF, and the proportions of the cells positive for green fluorescence (S/G_2_/M phase) and red fluorescence (G_1_ phase) were measured by flow cytometry. The proportion of green cells increased 24 h after RANKL stimulation, but this increase disappeared 48 h after stimulation (Fig. 1, *A* and *B*). M-CSF alone did not significantly influence the proportion of green or red cells during this period (Fig. 1, *A* and *B*). These results suggested that RANKL stimulation transiently promotes cell cycle progression. The increase in cell cycle progression was confirmed by BrdU incorporation assay (Fig. 1*C*). Using time-lapse imaging, we measured the doubling time of dTg-BMMs cultured with M-CSF in the presence or absence of RANKL for 48 h. RANKL significantly reduced doubling time in a dose-dependent manner (Fig. 1*D*), along with increasing formation of multinucleated osteoclasts (Fig. 1*E*). These results support the idea that RANKL stimulation increases the basal proliferation of dTg-BMMs.

##### RANKL Stimulation Induces Polyploid Cells Not Only by Cell Fusion but Also by Incomplete Cytokinesis

**FIGURE 2.**
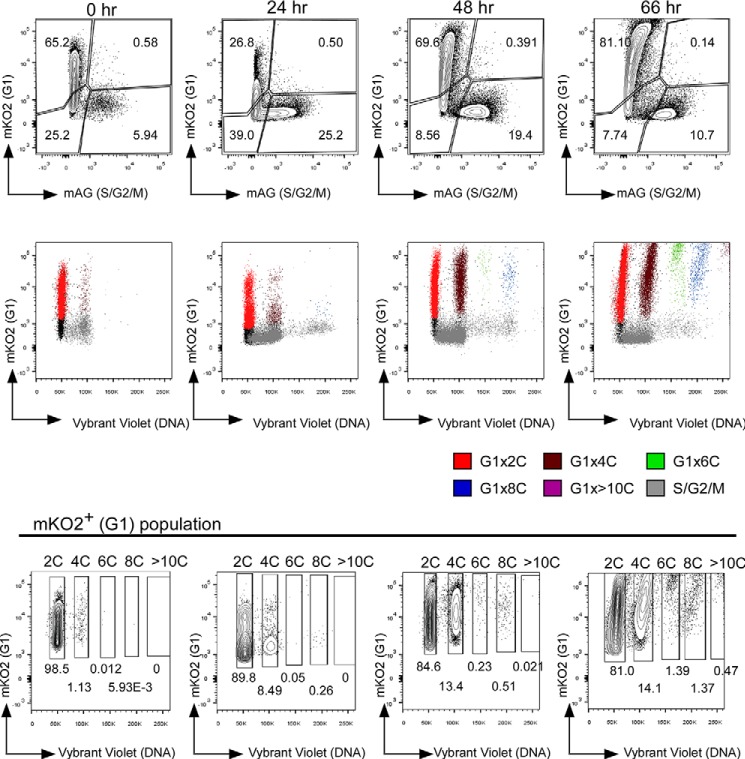
**RANKL induces polyploid cells.** Ploidy analysis of dTg-BMMs during osteoclast differentiation. dTg-BMMs were cultured with M-CSF (60 ng/ml) and RANKL (150 ng/ml) for the indicated times. Cells were harvested at the indicated times, stained with DNA staining dye (Vybrant DyeCycle Violet), and examined by flow cytometry. *Top row* shows flow cytometry results of dTg-BMMs cultured with RANKL for the indicated times. *Middle row* shows flow cytometry results of red fluorescence (mKO2) *versus* Vybrant DyeCycle Violet. *Bottom row* provides quantitation of the flow cytometry results shown above. 2C, 4C, 6C, 8C, and >10C cells are gated. *Numbers* indicate the percentages of *red* (mKO2) positive cells in each bin. Results are representative of three independent experiments.

We next performed ploidy analysis during osteoclast formation. dTg-BMMs were stimulated with RANKL for the indicated times in the presence of M-CSF, and ploidy was analyzed by flow cytometry (Fig. 2). As expected, stimulation with RANKL induced generation of polyploid cells (red fluorescence-positive 4C, 6C, 8C, and >10C) (Fig. 2). Among these polyploid cells, 4C and 8C cells were detected first after RANKL stimulation for 24 h (Fig. 2). By contrast, 6C cells were not detected until 48 h after the onset of RANKL stimulation, and 6C cells were less common than 8C cells (Fig. 2 and Table 1).

**TABLE 1:** **Percentage of diploid and polyploid cells among mKO2^+^ cells** Each row represents the result of an independent flow cytometry experiment.

	2C	4C	6C	8C	>10C
Monocytes	98.32	1.00			
Monocytes	97.93	1.20	0.03		
Monocytes	94.25	3.89			
Monocytes	92.42	5.36	0.04	0.08	
Monocytes	93.39	4.11	0.09	0.09	
OC (day2)	84.77	12.94	0.25	0.49	0.02
OC (day2)	83.17	13.04	0.17	0.73	
OC (day2)	77.27	16.02	0.66	1.27	
OC (day3)	77.92	13.10	1.32	1.47	1.61
OC (day3)	81.81	13.80	0.59	1.13	

**FIGURE 3.**
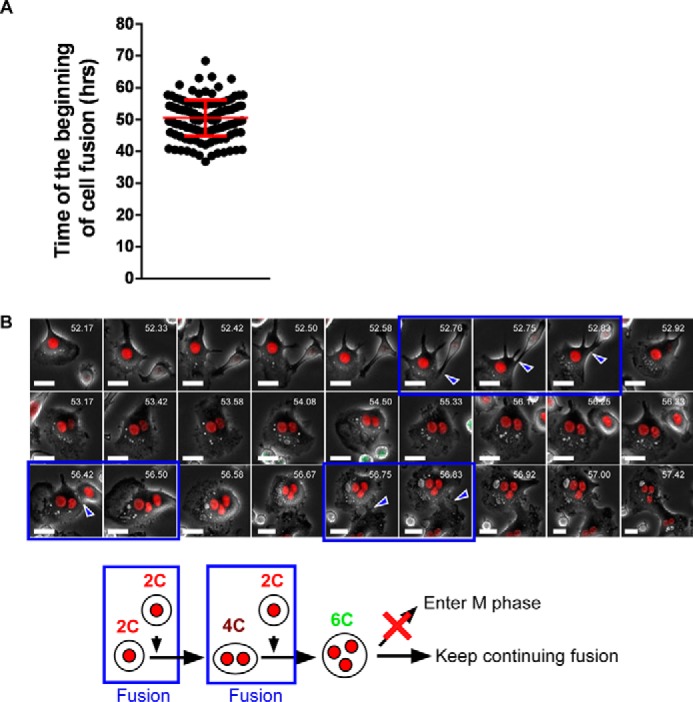
**Cell fusion occurs at and after 36 h of RANKL stimulation.** *A,* time of initiation of cell fusion after RANKL stimulation. Images were taken every 5 min, and the time required for cell fusion was calculated. A total of 145 cells that underwent cell fusion are plotted. *Red bar* indicates means ± S.D. *B,* representative snapshots of mononucleated cell-cell fusion. Fluorescence and phase-contrast images were taken every 5 min. *Blue arrowheads* and *boxes* indicate cell fusion. *White numbers* in each image indicates time after RANKL stimulation. *Bottom* schematically depicts the results observed in *B. Scale bars,* 20 μm.

**FIGURE 4.**
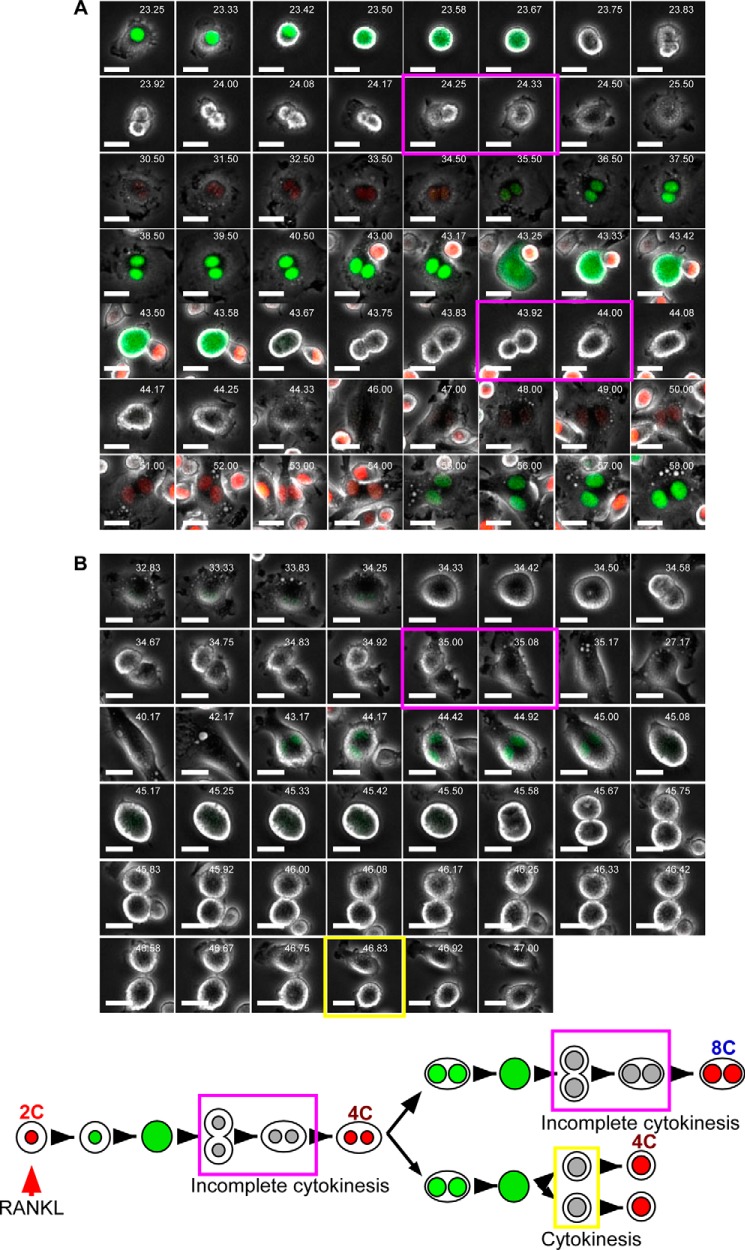
**RANKL stimulation induces formation of polyploid cells by incomplete cytokinesis.** *A,* representative snapshots of dTg-BMMs that underwent incomplete cytokinesis after RANKL stimulation. Fluorescence and phase-contrast images were taken every 5 min. *B,* representative snap shots of dTg-BMMs that underwent cytokinesis after incomplete cytokinesis. *Magenta boxes* indicate incomplete cytokinesis, and the *yellow box* indicates cytokinesis. *White numbers* in each image indicates time after RANKL stimulation. *Scale bars,* 20 μm. Results are representative of five independent experiments. *Bottom* shows the scheme depicting the results observed in *A* and *B*.

To examine how these polyploid cells were generated, we performed time-lapse imaging. In these experiments, dTg-BMMs were stimulated by RANKL for various times (1–16 h) in the presence of M-CSF, and then cell cycle progression and polyploidization were observed by time-lapse imaging. Regardless of the period of RANKL stimulation, almost all cells were mononucleated at the beginning of imaging. No cell fusion was observed within 24 h, but fusion was observed at and after 36 h of RANKL stimulation (average time of the beginning of fusion was 50.5 ± 5.63 h; Fig. 3*A*). The majority of cells that went through cell fusion were red fluorescence-positive mononucleated cells (Fig. 3*B* and Table 2), and the resultant fused cells rarely went through mitosis (Table 2). Instead, they continued to undergo cell fusion, and finally became large multinucleated osteoclast-like cells with red nuclei. These observations suggested that 4C and 8C cells detected after 24 h of RANKL stimulation were not fusion products. Unexpectedly, incomplete cytokinesis was observed during this period (Fig. 4*A* and supplemental Movie 1). The incomplete cytokinesis resulted in formation of 4C binucleated cells (Fig. 4*A*). Some of these cells re-entered mitosis but failed to complete cytokinesis, resulting in formation of binucleated 8C cells (Fig. 4*A*). Consistent with this, after RANKL stimulation for 24 h, flow cytometric analysis detected S/G_2_/M phase cells between the 4C and 8C peaks (Fig. 2), consistent with the observation that some of the tetraploid cells re-entered the cell cycle. Together, these observations suggested that 4C and 8C cells detected at 24 h after RANKL stimulation resulted from incomplete cytokinesis.

**TABLE 2:** **Fusion summary of osteoclasts**

Event	Fusion combination			
	mKO2 (DN) × mKO2 (DN)	mKO2 (DN) × mAG	mAG × mAG	Total no. (%)
Cell fusion	132	12	1	145
Cell fusion involving incomplete cytokinesis	16	0	0	16 (11.0)
Cytokinesis after fusion	0	0	1	1 (0.69)

##### Cells That Undergo Incomplete Cytokinesis Have the Potential for Cell Fusion

**FIGURE 5.**
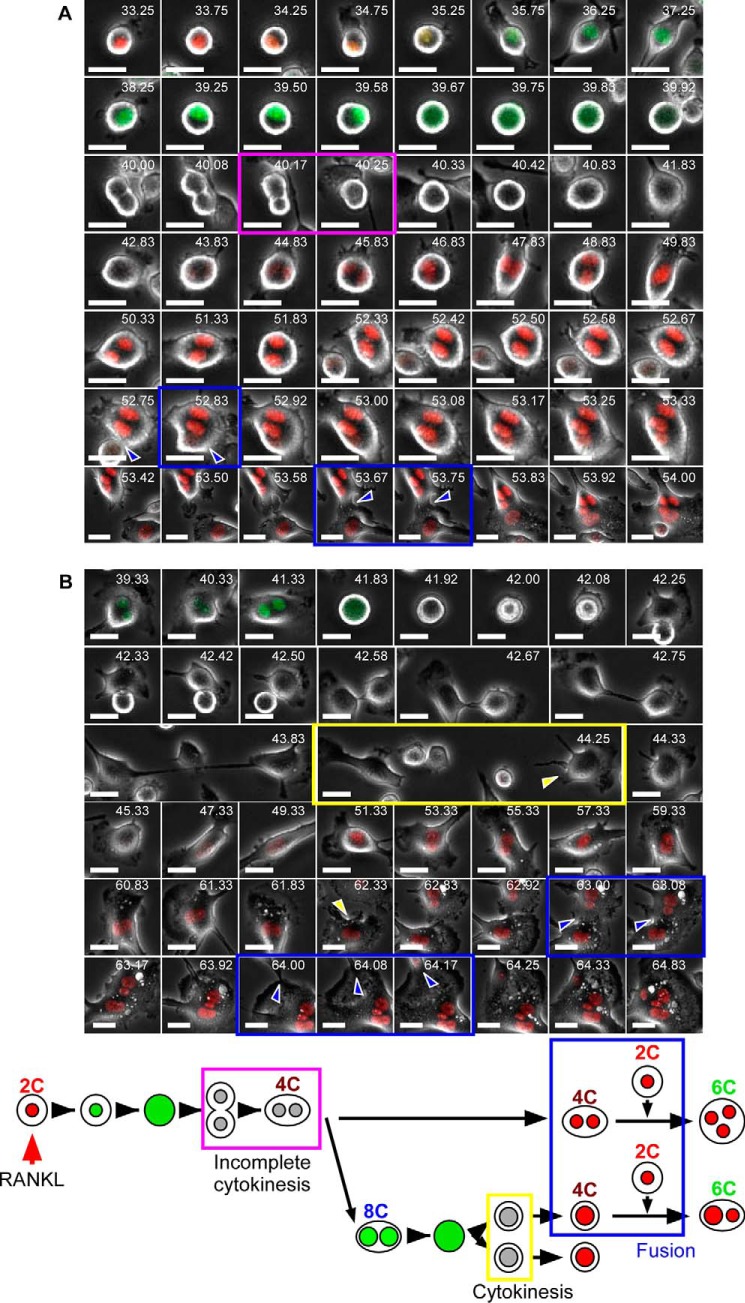
**Cells that undergo incomplete cytokinesis have the potential to undergo cell fusion.** *A,* representative snapshots of a binucleated cell that underwent incomplete cytokinesis and subsequently underwent cell fusion. Fluorescence and phase-contrast images were taken every 5 min. *B,* representative snapshots of a mononucleated cell that underwent incomplete cytokinesis and subsequently underwent cell fusion. *Blue arrowheads* and *blue boxes* indicate cell fusion. The *magenta box* indicates incomplete cytokinesis, and the *yellow box* indicates cytokinesis. *White numbers* in each image indicate the time after RANKL stimulation. *Scale bars,* 20 μm. Results are representative of five independent experiments. *Bottom* shows the scheme depicting the results observed in *A* and *B*.

Because generation of polyploidy by incomplete cytokinesis has been observed in certain pathological contexts (25, 26), we speculated that cells that undergo incomplete cytokinesis might merely be abnormal, rather than critical intermediates in the formation of mature osteoclasts. To address this issue, we continued time-lapse imaging to trace the fates of cells that underwent incomplete cytokinesis. Cell-tracking analysis revealed that binucleated cells generated by incomplete cytokinesis could undergo cell fusion (Fig. 5*A* and supplemental Movie 2). Indeed, 11% of fused cells had previously undergone incomplete cytokinesis (Table 2). These results indicated that such cells had the potential to undergo cell fusion during RANKL-induced osteoclast formation.

Some of the binucleated cells generated by incomplete cytokinesis ultimately succeeded in undergoing cytokinesis and formed mononucleated polyploid cells (Fig. 4*B* and supplemental Movie 3). These mononucleated polyploid cells also had the potential to undergo cell fusion (Fig. 5*B* and supplemental Movie 4). Notably, ∼50% of 4C cells sorted from dTg-BMMs cultured with RANKL for 66 h were mononucleated.^4^ These observations strongly suggested that mononucleated cells involved in cell fusion consisted of both 2C and polyploid cells. During time-lapse imaging, target cells often migrated away from the field of view. In this case, the fate of the target cells could not be traced, and consequently those cells were uncounted. Thus, the observed percentage of cell fusion involving cells that undergo incomplete cytokinesis might represent an underestimate. Collectively, these results revealed that polyploid cells detected after RANKL stimulation for more than 48 h consisted not only of fusion products but also of cells that underwent incomplete cytokinesis.

##### Characterization of Cells That Undergo Incomplete Cytokinesis

**FIGURE 6.**
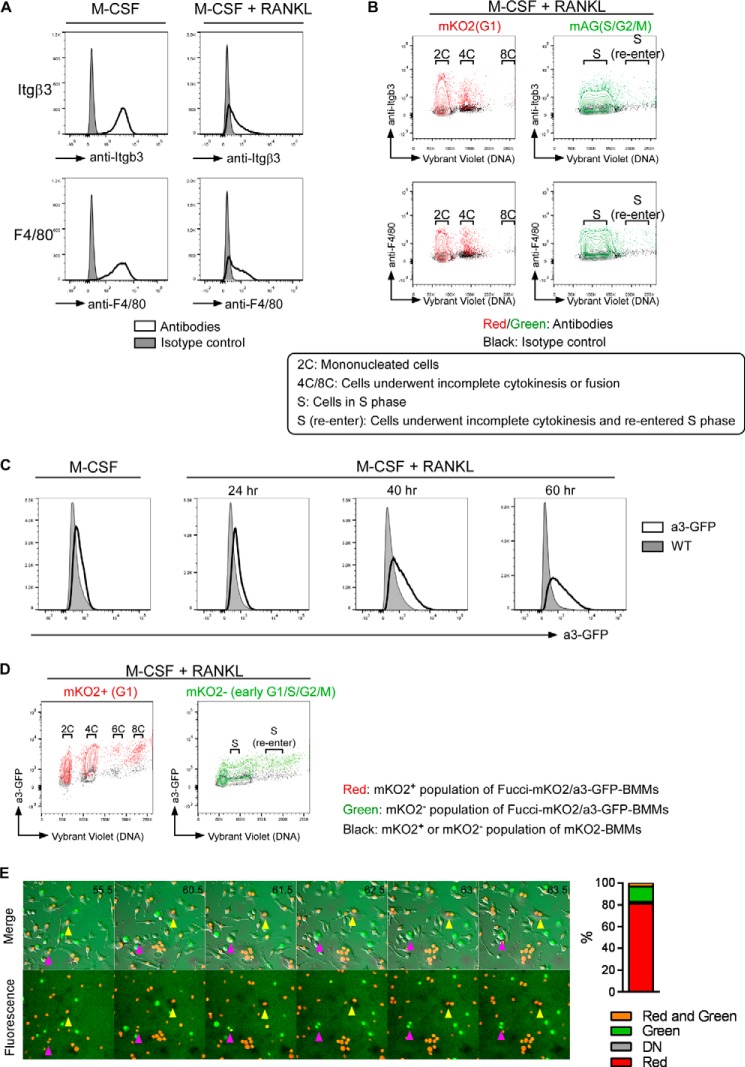
**Characterization of cells that undergo incomplete cytokinesis.** *A,* dTg-BMMs were cultured with M-CSF (60 ng/ml) in the presence or absence of RANKL (150 ng/ml). After 48 h of culture, cells were harvested and stained with anti-integrin β3 or anti-F4/80. The levels of these molecules were determined by flow cytometry. *B,* dTg-BMMs described in *C* were divided into four groups depending on their cell cycle state and DNA contents as follows: mononucleated cells (*G1* × *2C*); cells in S phase (*S*); cells that underwent incomplete cytokinesis or were generated by cell fusion (*G1* × *4C, G1* × *8C*); and cells that underwent incomplete cytokinesis and re-entered S phase (*S* (*re-enter*)). *C,* expression profiles of a3-GFP in Fucci-mKO2/a3-GFP-BMMs cultured with M-CSF (60 ng/ml) in the presence or absence of RANKL (150 ng/ml) for the indicated times. *D,* Fucci-mKO2/a3-GFP-BMMs cultured with M-CSF (60 ng/ml) and RANKL (150 ng/ml) for 60 h were divided into diploids, polyploids, and cells in S phase (*S* (*re-enter*)) depending on their cell cycle state and DNA contents. *E, left,* representative snapshots of dTg-BMMs cultured on FITC gelatin-coated culture dishes. dTg-BMMs cultured on FITC gelatin-coated dishes for 24 h were observed. Fluorescence and bright field images were taken every 30 min. *Yellow arrowheads* indicate a cell in G_1_ phase that underwent cell fusion, and *magenta arrowheads* indicate a cell that underwent incomplete cytokinesis and re-entered S phase (*S* (*re-enter*)). *Black numbers* in each image indicate time after RANKL stimulation (hours). *Right,* percentage of cells that degraded FITC gelatin during the imaging.

To understand the phenotype of cells that had undergone incomplete cytokinesis, we first examined the expression profiles of integrin β3, F4/80, and V-type H^+^-ATPase a3 subunit in RANKL-stimulated BMMs. Integrin β3 is an important cell adhesion molecule that plays an important role in osteoclast biology (27), and F4/80 is a macrophage marker that is barely expressed on osteoclasts (28). Expression of both integrin β3 and F4/80 was detected on dTg-BMMs and changed after 48 h of stimulation with RANKL (Fig. 6*A*). We separated dTg-BMMs into four groups depending on their cell cycle phase and DNA content as follows: (i) mononucleated cells (G_1_ ×2C); (ii) cells in S phase (S); (iii) cells that underwent incomplete cytokinesis or were generated by cell fusion (G_1_ ×4C, G_1_ × 8C), and (iv) cells that underwent incomplete cytokinesis and re-entered S phase (*S* (*re-enter*)) (Fig. 6*B*). The expression of integrin β3 and F4/80 was examined in each of these groups. Flow cytometry analysis revealed that the expression levels of these molecules were approximately the same in all four groups (Fig. 6*B*), suggesting that the expression of integrin β3 and F4/80 was not affected by incomplete cytokinesis.

V-type H^+^-ATPase a3 subunit is a protein complex that mediates H^+^ transport into the resorption lacunae. Lack of a3 subunit results in severe osteopetrosis due to impaired osteoclast bone-resorbing function (29). BMMs derived from Fucci-mKO2 and a3-GFP-expressing mice (Fucci-mKO2/a3-GFP-BMMs) were cultured with M-CSF and RANKL for the indicated times, and the expression of a3-GFP was analyzed by flow cytometry. The expression of a3-GFP gradually increased with time (Fig. 6*C*) and was detected in diploids (G_1_ × 2C), polyploids (G_1_ × 4C, G_1_ × 6C, and G_1_ × 8C), and cells that underwent incomplete cytokinesis and re-entered S phase (*S* (*re-enter*)) (Fig. 6*D*). These results suggested that the expression of a3-GFP was not affected by incomplete cytokinesis.

We next examined resorption activities of cells that had undergone incomplete cytokinesis. dTg-BMMs were cultured on FITC-labeled gelatin-coated dishes in the presence of M-CSF and RANKL for 24 h, and then time-lapse imaging was performed. Resorption activity can be observed as degradation of the gelatin. Majority of cells that degraded FITC gelatin were in red (81.0%; Fig. 6*E*). In contrast, green^+^ binucleated cells (*i.e.* the cells that underwent incomplete cytokinesis and re-entered S phase) barely degraded FITC gelatin (Fig. 6*E* and supplemental movie 5). These results suggested that cells that had undergone incomplete cytokinesis and re-entered S phase had lower resorption capacity than cells in G_1_ phase.

##### Cells That Undergo Incomplete Cytokinesis Are Involved in Formation of Multinucleated Osteoclasts in Vivo

**FIGURE 7.**
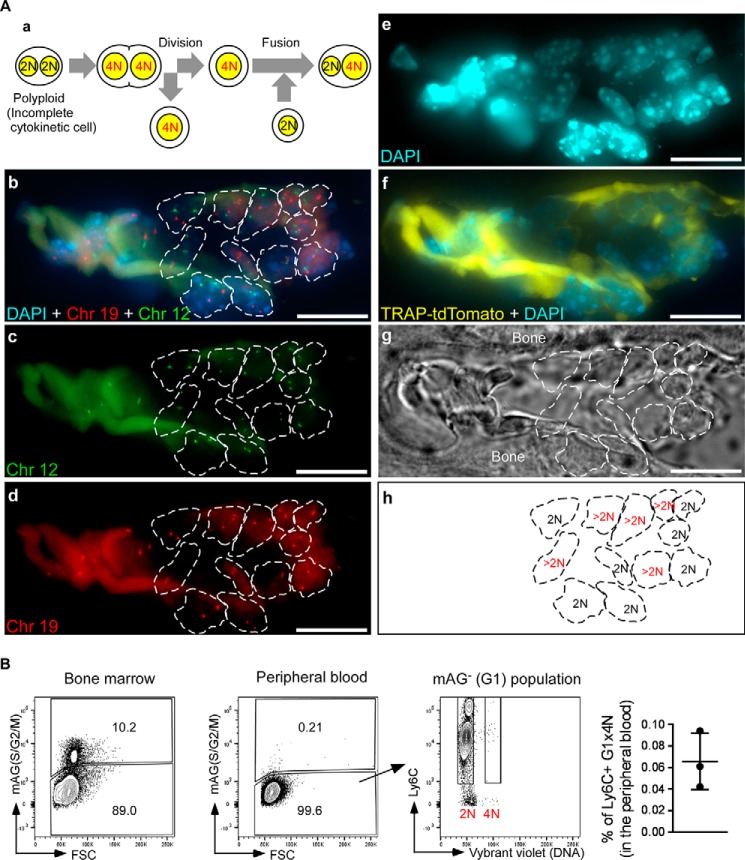
**Diploid and polyploid nuclei are observed in osteoclasts *in vivo*.** *A,* representative micrographs of fluorescence *in situ* hybridization (FISH). *A, panel a,* scheme depicting generation of polyploidy by cell fusion of diploid cells and cells that undergo incomplete cytokinesis. *Panel b,* bone sections of TRAP promoter-tdTomato transgenic mice were hybridized with probes for chromosome (*Chr*) 12 (*green*) and chromosome 19 (*red*). Nuclei were stained with DAPI (*blue*). *Panel c,* micrograph of FISH with a probe for chromosome 12 in the same visual field as *panel b. Panel d,* micrograph of FISH with a probe for chromosome 19 in the same visual field as *panel b. Panel e,* micrograph of nuclei (DAPI: *blue*) in the same visual field as *panel b. Panel f,* merged micrograph of TRAP-tdTomato cells (*yellow*) and nuclei (DAPI: *blue*) in the same visual field as *panel b. Panel g,* a bright field micrograph of the same view filed shown in *panel b. Panel h,* outlines of nuclei in the micrographs shown above. Ploidy of each nucleus is written inside the corresponding outline. *Scale bars,* 10 μm. *B,* flow cytometry analysis of osteoclast precursors *in vivo*. Peripheral blood cells or bone marrow cells were prepared from Fucci-mAG mice and examined the cell cycle phase, expression of Ly6C, and DNA content. The *graph* shows percentage of Ly6C^+^ G_1_ × 4C cells in the peripheral blood. Each *circle* represents the result of a mouse. *Bars* indicate means ± S.D.

To determine whether incomplete cytokinesis plays a physiologically relevant role in development of multinucleated osteoclasts, we performed two experiments. First, we examined the ploidy of osteoclasts *in vivo*. Fusion of cells that had previously undergone incomplete cytokinesis would result in some osteoclasts exhibiting nuclear polyploidy (*i.e.* containing nuclei with more than the diploid complement of chromosomes) (Fig. 7*A, panel a*). In transgenic mice in which tdTomato, a red fluorescent protein, is expressed under the control of the TRAP promoter, osteoclasts are labeled with tdTomato. We analyzed the ploidy profiles of osteoclasts in the long bone of TRAP-tdTomato transgenic mice by fluorescence *in situ* hybridization. In tdTomato^+^ osteoclasts, some nuclei had the diploid complement of chromosomes (2N), whereas others had more than the diploid complement (>2N) (Fig. 7*A*, *panels b–h*).

Next, we examined the existence of osteoclast precursors that had undergone incomplete cytokinesis *in vivo* by flow cytometry. Because Ly6C is reported as one of markers of osteoclast precursors (30), and osteoclast precursors are circulating in the periphery (31), we tried to detect Ly6C^+^ G_1_ × 4C cells in the peripheral blood. In the peripheral blood of Fucci-mAG transgenic mice, green^+^ cells were barely detected. In green^−^ (G_1_ phase) cells, Ly6C^+^ 4C cells were detected (average percentage was 0.07 ± 0.03%; Fig. 7*B*). These results indicated that almost all circulating cells were in G_1_ phase under physiological conditions, and G1 × 4C osteoclast precursors could be detected in the periphery. Although we cannot exclude the possibility that the cells were binucleated fusion products, this observation may support in some part the idea that incomplete cytokinesis occurs *in vivo*. These results suggested that fusion of cells that undergo incomplete cytokinesis occurs *in vivo* and that incomplete cytokinesis contributes to physiological development of multinucleated osteoclasts.

##### Fusion of Cells That Undergo Incomplete Cytokinesis Occurs during Formation of Osteoclasts but Not MGCs

**FIGURE 8.**
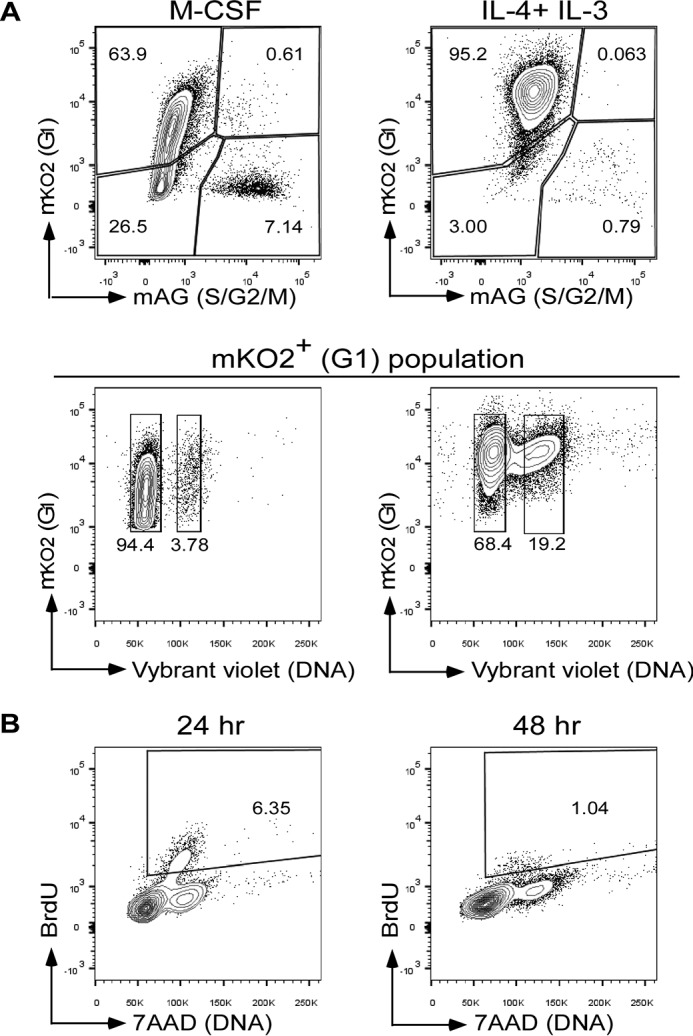
**Flow cytometry analysis of dTg-BMMs during MGC formation.** *A, top,* dTg-BMMs were cultured with IL-4 (100 ng/ml) plus IL-3 (100 ng/ml) for 48 h to induce formation of MGCs. Cells were harvested, and cells positive for red (mKO2) or green (mAG) fluorescence were detected by flow cytometry. *Bottom*, ploidy analysis of dTg-BMMs during MGC differentiation. dTg-BMMs cultured with IL-4 (100 ng/ml) and IL-3 (100 ng/ml) for 48 h were harvested, stained with DNA staining dye (Vybrant DyeCycle Violet), and examined by flow cytometry. 2C and 4C cells of *red* (mKO2) fluorescence-positive cells are gated. *Numbers* indicate the percentages of *red* (mKO2)-positive cells in each bin. Results are representative of three independent experiments. *B,* BrdU incorporation assay. WT-BMMs were cultured with IL-4 (100 ng/ml) plus IL-3 (100 ng/ml) for the indicated amount of time (in hours). BrdU (10 μm) was added for the last 6 h. Cells were harvested, and incorporated BrdU was stained with FITC-labeled anti-BrdU antibody. DNA was stained with 7-AAD and analyzed by flow cytometry. *Numbers* indicate the percentages of S phase cells. Results are representative of three independent experiments.

Next, we examined the involvement of incomplete cytokinesis in formation of MGCs (32, 33). MGCs are formed by cell fusion of macrophages in response to foreign bodies at the site of implantation, and they can be formed *in vitro* from monocytes following stimulation with combinations of cytokines such as IL-4 and IL-3. Various common molecules (*e.g.* DC-STAMP, OC-STAMP, and Atp6v0d2) are required for polyploidization of both osteoclasts and MGCs (13, 34, 35), suggesting that MGCs might become multinuclear via a process similar to that of osteoclasts. Stimulation with IL-4 plus IL-3 induced formation of polyploid cells but only rarely induced cell proliferation (Fig. 8*A*). This observation was confirmed by BrdU incorporation assays (Fig. 8*B*). Consistent with these observations, neither incomplete cytokinesis nor cell fusion involving incomplete cytokinesis was observed (Table 3). These results suggested that fusion of cells that undergo incomplete cytokinesis may be involved in formation of RANKL-induced osteoclasts but not MGCs.

**TABLE 3:** **Fusion summary of MGCs**

Event	Fusion combination			
	mKO2 (DN) × mKO2 (DN)	mKO2 (DN) × mAG	mAG × mAG	Total no. (%)
Cell fusion	64	12	0	76
Cell fusion involving incomplete cytokinesis	0	0	0	0 (0.0)
Cytokinesis after fusion	2	1	0	3 (3.95)

**FIGURE 9.**
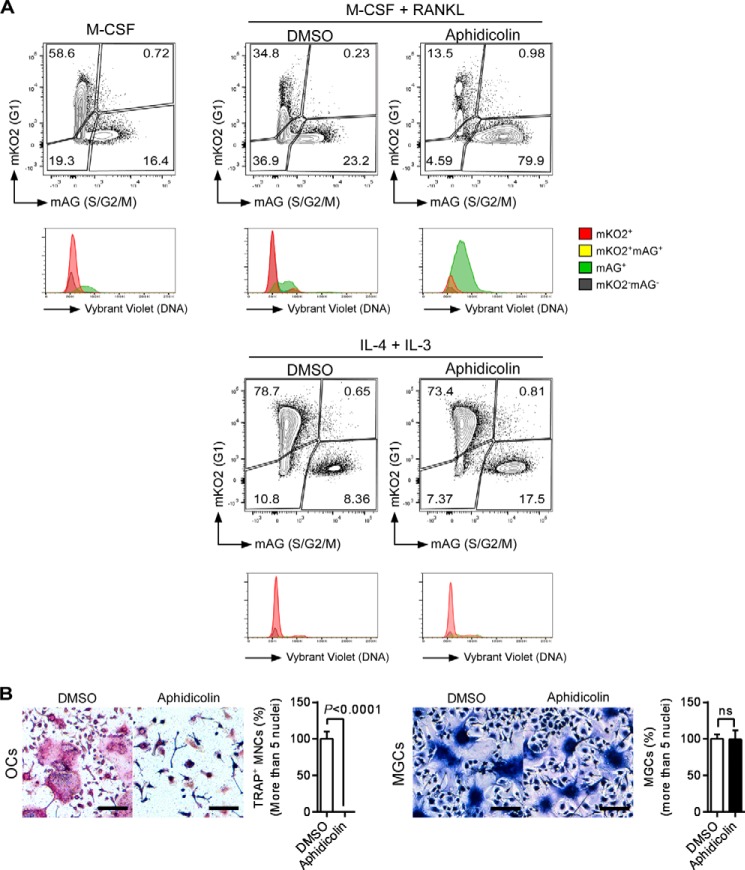
**Flow cytometry analysis of dTg-BMMs after treatment with cell cycle inhibitors.** *A,* dTg-BMMs were stimulated with M-CSF (60 ng/ml) and RANKL (150 ng/ml) or IL-4 (100 ng/ml) plus IL-3 (100 ng/ml) in the presence of aphidicolin (500 nm) or DMSO for 24 h. Cells were harvested and analyzed by flow cytometry. Results are shown in contour plots and histograms. *B,* TRAP staining (osteoclasts) or Giemsa staining (MGCs) is shown. Percentage of multinucleated cells containing more than five nuclei. Data represent means ± S.D. Results are representative of three independent experiments. *OC*, osteoclasts; *ns*, not significant.

Cell cycle progression seems to be required for formation of multinucleated osteoclasts but not MGCs. To address this point, we examined the effect of blocking cell cycle on multinucleation of osteoclasts and MGCs. Treatment with aphidicolin, an inhibitor of nuclear DNA replication that blocks the cell cycle at early S phase, drastically increased green cell proportion in RANKL-stimulated osteoclasts, but only slightly in IL-4 +IL-3-stimulated MGCs (Fig. 9*A*), indicating that aphidicolin induced S phase arrest in osteoclasts but not in MGCs. In addition, treatment with aphidicolin significantly inhibited formation of multinucleated osteoclasts, but not MGCs (Fig. 9*B*). These results suggested that RANKL but not IL-4 plus IL-3 promoted cell cycle progression and supported the idea that cell cycle progression is necessary for formation of multinucleated osteoclasts but not MGCs. Therefore, although osteoclasts and MGCs are generated from the same lineage of progenitors and require common molecules to become multinuclear, the mechanisms underlying the formation of these distinct types of multinucleated cells are likely to be different.

##### RANKL-induced Akt Activation Controls Incomplete Cytokinesis during Osteoclast Development

**FIGURE 10.**
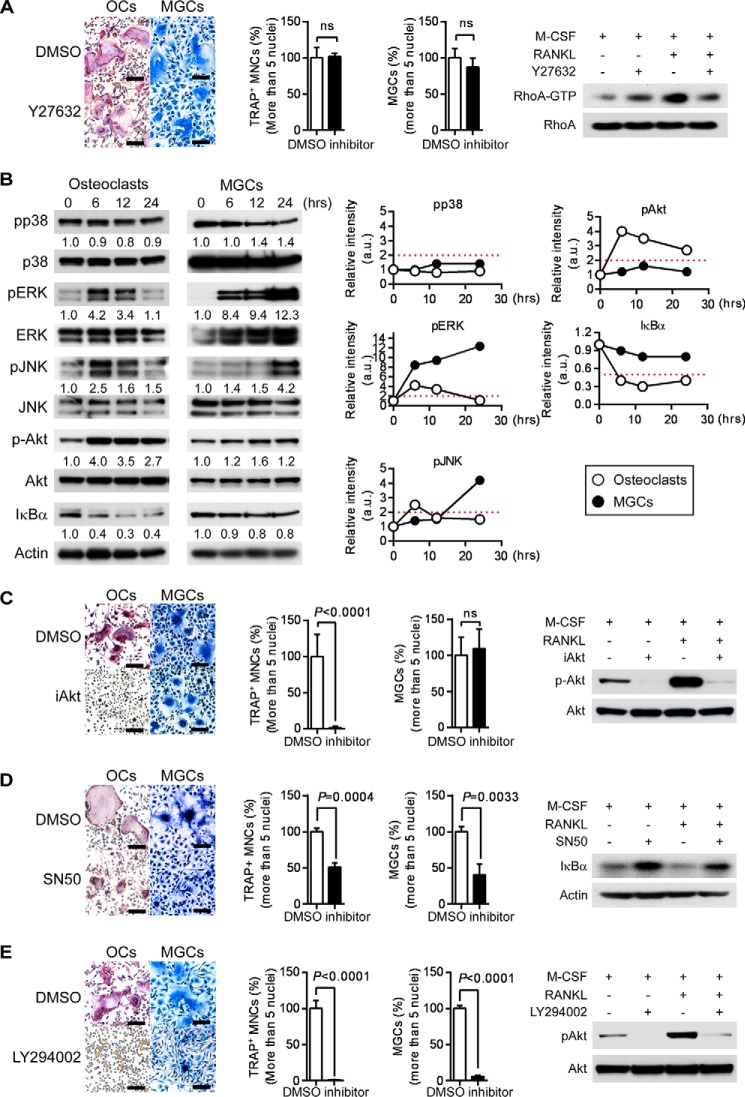
**Searching signaling molecule(s) that regulates osteoclast multinucleation.** *A, left, in vitro* differentiation of osteoclasts or MGCs from BMMs treated with Y27632 (10 μm) or DMSO. TRAP staining (osteoclasts) or Giemsa staining (MGCs) is shown. Percentage of multinucleated cells containing more than five nuclei. Data represent means ± S.D. *Right,* BMMs were pretreated for 2 h with Y27632 or DMSO and then stimulated with or without RANKL in the presence of M-CSF for 6 h. Cell lysates were used for RhoA activation assay and immunoblotted with the indicated antibodies. *B,* phosphorylation of MAPKs and Akt and degradation of IκBα during osteoclast or MGC differentiation. Lysates from BMMs stimulated with M-CSF (60 ng/ml) plus RANKL (150 ng/ml) (osteoclasts) or IL-4 (100 ng/ml) plus IL-3 (100 ng/ml) (MGCs) for the indicated times were immunoblotted with the indicated antibodies. Relative band intensities are shown below the Western blot (*left*) and plotted (*right*). *Red dashed lines* indicate relative signal intensities twice as much as those in monocytes (0 h). *a.u.,* arbitrary unit. *C–E, left, in vitro* differentiation of osteoclasts or MGCs from BMMs treated with Akt inhibitor VIII (iAkt, 5 μm) (*C*), SN50 (50 μg/ml) (*D*), LY294002 or DMSO (5 μm) (*E*). TRAP staining (osteoclasts) or Giemsa staining (MGCs) is shown. Percentage of multinucleated cells containing more than five nuclei. Data represent means ± S.D. *Right,* BMMs were pretreated for 2 h with Akt inhibitor VIII, SN50, LY294002, or DMSO and then stimulated with or without RANKL in the presence of M-CSF for 6 h. Cell lysates were immunoblotted with the indicated antibodies. Percentage of multinucleated cells containing more than five nuclei. Data represent means ± S.D. Results are representative of three independent experiments. *OC*, osteoclasts; *ns,* not significant.

To understand the molecular mechanism by which incomplete cytokinesis is controlled during RANKL-induced osteoclast multinucleation, we first examined a small GTPase, Rho, which plays a role in cytokinesis by regulating the contractile ring (36, 37). Treatment with Y27632, a selective inhibitor of Rho-associated protein kinase (ROCK), a downstream target of Rho, neither inhibited nor promoted generation of multinucleated osteoclasts (Fig. 10*A*). These results suggested that Rho is not an important factor in RANKL-induced osteoclast multinucleation.

We next searched for signal molecules whose activation is induced in osteoclasts but not in MGCs. We examined phosphorylation of MAPKs (p38, ERK, and JNK) and Akt and degradation of IκBα. We found that phosphorylation of Akt and degradation of IκBα were induced in RANKL-stimulated osteoclasts more than twice as much as those in monocytes but were only slightly induced in IL-4 + IL-3-stimulated MGCs (Fig. 10*B*). Both Akt and NF-κB are involved in osteoclast formation (38–40). Consistent with previous reports, blocking Akt activation by treatment with Akt inhibitor VIII or blocking NF-κB activation by treatment with SN50 prevented RANKL-induced formation of multinucleated osteoclasts (Fig. 10, *C* and *D*). Blocking NF-κB activation also inhibited MGC formation (Fig. 10*D*). However, blocking of Akt activation did not inhibit IL-4 + IL-3-induced MGC formation (Fig. 10*C*).

**FIGURE 11.**
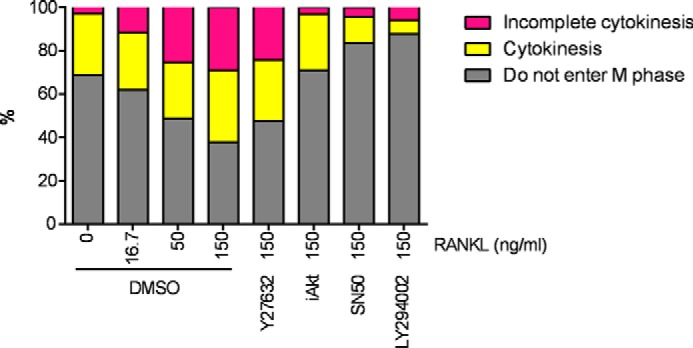
**Akt regulates incomplete cytokinesis and formation of multinucleated osteoclasts.** *A,* percentage of cells that went through incomplete cytokinesis (*magenta*) or cytokinesis (*yellow*). dTg-BMMs were cultured with M-CSF (60 ng/ml) and RANKL in the presence of the indicated reagents. Cells that did not enter mitotic phase are shown in *gray*. Data shown as percentage of cells pooled from two independent experiments.

To better understand the role of Akt and NF-κB in multinucleation and incomplete cytokinesis of osteoclasts, we performed time-lapse imaging. In these experiments, dTg-BMMs were stimulated with RANKL in the presence or absence of inhibitors for 1 h, and then cell cycle progression and polyploidization were observed. RANKL induced incomplete cytokinesis in a dose-dependent manner (Fig. 11), and the increase in incomplete cytokinesis was significantly inhibited when Akt activation was blocked (89.3% lower than in control osteoclasts treated with 150 ng/ml RANKL; Fig. 11). Akt inhibitor VIII also decreased entry to mitotic phase by half (53.4% lower than in control osteoclasts treated with 150 ng/ml RANKL; Fig. 11), suggesting that Akt may play a role in regulating both incomplete cytokinesis and cell cycle progression during osteoclast differentiation. Inhibition of NF-κB activation by SN50 decreased not only incomplete cytokinesis (85.2% lower than in control osteoclasts treated with 150 ng/ml RANKL; Fig. 11) but also entry to mitotic phase (73.5% lower than in control osteoclasts treated with 150 ng/ml RANKL; Fig. 11), suggesting that NF-κB signaling pathway is involved in cell cycle progression in osteoclasts. Considering that SN50 inhibited multinucleation of MGCs (Fig. 10*D*), which were generated by incomplete cytokinesis-independent fusion (Figs. 8 and 9), it is plausible that NF-κB signaling pathway plays roles not only in cell cycle progression but also in fusion. Taken together, these results suggest that Akt-dependent RANKL-induced incomplete cytokinesis is involved in the formation of polyploid osteoclasts.

## Discussion

Osteoclasts require polyploidization to acquire sufficient bone-resorbing activity. Using Fucci-expressing BMMs, we were able to distinguish polyploid cells from S/G_2_/M phase cells by flow cytometry. In combination with time-lapse imaging analysis, we showed that the polyploidization of osteoclasts is due to not only cell fusion, but also incomplete cytokinesis. In addition, we demonstrated nuclear polyploidy of osteoclasts *in vivo*. Although incomplete cytokinesis has been extensively studied in pathological contexts, its role in physiological contexts remains unclear. Our findings reveal the importance of incomplete cytokinesis in the formation of polyploid osteoclasts, a normal physiological process.

Using inhibitors, we sought to identify signaling molecules that control incomplete cytokinesis during osteoclast polyploidization. Blocking ROCK neither inhibited nor promoted osteoclast multinucleation and had no effect on cytokinesis or incomplete cytokinesis during osteoclast formation (Figs. 10*A* and 11). Although the Rho signaling pathway regulates cytokinesis by driving actin and myosin, our observations suggested that it may not play a major role in either cytokinesis/incomplete cytokinesis or cell fusion in osteoclast polyploidization. Blocking NF-κB decreased formation of osteoclasts and MGCs (Fig. 10, *B* and *D*). In addition, blocking NF-κB activation inhibited not only incomplete cytokinesis but also cell cycle progression (Fig. 11). These results suggested that NF-κB may not be specifically involved in regulation of incomplete cytokinesis.

We identified Akt as a key molecule involved in the control of incomplete cytokinesis during osteoclast polyploidization. Notably in this regard, Akt plays a role in the formation of tetraploid vascular smooth endothelial cells and hepatocytes by regulating incomplete cytokinesis (41, 42). Our results suggest that Akt-mediated incomplete cytokinesis is a general program involved in the formation of polyploidy. We cannot exclude the possibility that Akt also plays a role in the cell fusion process. Future work should further explore the role of Akt in incomplete cytokinesis and cell fusion. Blocking Akt activation also attenuated entry into mitosis (Fig. 11). Indeed, Akt is involved in cell cycle regulation (43). These observations suggest that Akt plays multiple roles during osteoclastogenesis, including regulation of both incomplete cytokinesis and cell cycle progression. Of note, stimulation with RANKL, but not IL-4 plus IL-3, promoted cell cycle progression (Fig. 9*A*), and blocking the RANKL-induced cell cycle progression inhibited formation of multinucleated osteoclasts (Fig. 9*B*). These results reinforce the idea that mitotic entry and cell cycle progression are important for generation of multinucleated osteoclasts.

Akt is an important downstream effector of PI3K, which is also involved in osteoclast formation (44), suggesting that the PI3K-Akt signaling pathway plays a role in osteoclast multinucleation. However, blocking of PI3K activation by treatment with the selective PI3K inhibitor LY294002 significantly inhibited not only incomplete cytokinesis (79.5% lower than in control-treated osteoclasts) but also cytokinesis (81.3% lower than in control-treated osteoclasts) (Fig. 11). In addition, blocking PI3K inhibited formation of both RANKL-induced multinucleated osteoclasts and IL-4 + IL-3-induced MGCs (Fig. 10*E*). In addition to Akt, PI3K also regulates Vav3, phospholipase Cγ2, and Grb2 activation (45). Hence, it is plausible that PI3K regulates a number of biological events, including incomplete cytokinesis. Further investigation will be required to understand the contribution of the PI3K-Akt pathway to regulation of RANKL-induced incomplete cytokinesis. We also examined the involvement of molecules previously determined to play a role in cell fusion, such as DC-STAMP, in blocking antibody experiments (anti-DC-STAMP mAb, clone 1A2); however, we did not see a specific effect on incomplete cytokinesis (data not shown). Future studies should attempt to clarify the involvement of fusion-related molecules in incomplete cytokinesis.

The molecular mechanism that selectively regulates incomplete cytokinesis during polyploidization of osteoclasts remains unclear. We observed Akt-regulated incomplete cytokinesis in RANKL-induced osteoclasts but not in IL-4 + IL-3-induced MGCs. These results suggest that the binding of RANKL to receptor activator of nuclear factor-κB, the receptor for RANKL, triggers signaling pathways that selectively regulate incomplete cytokinesis. Further studies will be required to determine the molecular mechanisms underlying regulation of incomplete cytokinesis by Akt during osteoclastogenesis.

**FIGURE 12.**
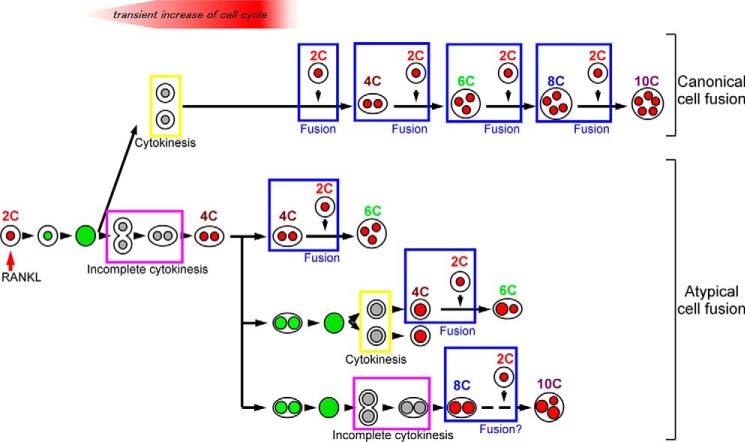
**Schematic model depicting the process of osteoclast polyploidization.** RANKL stimulation induces a transient increase in cell cycle activity, leading to cell fusion (canonical cell fusion). Along with the transient increase in cell cycle progression, some cells undergo incomplete cytokinesis. The resultant cells have the potential to undergo cell fusion and are involved in the formation of polyploid osteoclasts (atypical cell fusion).

We observed a low incidence (11%) of cell fusion involving incomplete cytokinesis during formation of multinucleated osteoclasts *in vitro* (Table 2), implying that there are at least two types of polyploid osteoclasts as follows: one generated by diploid cells (canonical cell fusion) and the other generated by diploid cells and cells that undergo incomplete cytokinesis (atypical cell fusion) (Fig. 12). We did not try to observe cell fusion following incomplete cytokinesis (atypical cell fusion) *in vivo* because the phenomenon takes more than a day, surpassing the current time limit for intravital imaging. Instead, we used fluorescence *in situ* hybridization to reveal that osteoclasts have nuclei with more than the diploid complement of chromosomes (>2N). In addition, we found circulating osteoclast precursors (Ly6C^+^ cells) that were G_1_ × 4C. These results suggested that cells that increase ploidy via cell cycle-dependent mechanisms are involved in the formation of multinucleated osteoclasts *in vivo*. However, we cannot exclude the possibilities that the nuclear polyploidy might also be caused by nuclear fusion, and Ly6C^+^ G_1_ × 4C cells were merely fusion products. Further studies will be required to clarify this issue.

The proportion of multinucleated osteoclasts generated by atypical cell fusion *in vivo*, as well as the functional differences between osteoclasts generated by canonical cell fusion and osteoclasts generated by atypical cell fusion, still remains unclear. FITC gelatin resorption assay showed that the cells that underwent incomplete cytokinesis and re-entered S phase (green^+^ binucleated cells) barely degraded FITC gelatin (Fig. 6*E*). These observations suggested that osteoclasts generated by atypical cell fusion might have relatively lower resorption activity than that by canonical cell fusion. However, it cannot be excluded that the difference of the resorption activity between cells in G_1_ phase and cells in S phase merely reflected the difference of their cell cycle phase. To address these issues, it will be necessary to identify specific markers expressed on cells that undergo incomplete cytokinesis.

What is the physiological significance of polyploid nuclei within a cell? This phenomenon may be just one aspect of the phenotype of terminally differentiated cells, or a consequence of stress response that preserves cell function. Alternatively, polyploid nuclei may create genetic diversity, which could promote better adaptation to chronic injury or stress (2, 46, 47). Very little is known regarding the physiological function of the polyploid state, largely due to technical limitations (*e.g.* there currently exist no methods for converting a tissue composed of polyploid cells into a tissue of the same size composed of diploid cells). A full understanding of the mechanisms of polyploidization is necessarily to reveal the physiological significance of osteoclast polyploidization via cell fusion and incomplete cytokinesis.

## Author Contributions

N. T. and Y. C. designed the study and wrote the paper. N. T., H. K., and H. M. performed and analyzed the experiments. A. S., A. M., M. T., O. K., and M. I. provided reagents and data analysis. All authors reviewed the results and approved the final version of the manuscript.

## Supplementary Material

Supplemental Data

## Footnotes

4 N. Takegahara and Y. Choi, unpublished observations.

## Footnotes

3 The abbreviations used are:

RANKL: receptor activator of nuclear factor-κB ligand
MGC: multinucleated giant cell
BMM: bone marrow-derived macrophage
dTg: double-transgenic
7-AAD: 7-aminoactinomycin
BP: band path filter
mAG: monomeric Azami Green.
