# Supplementary material for: Involvement of Receptor Activator of Nuclear Factor-κB Ligand (RANKL)-induced Incomplete Cytokinesis in the Polyploidization of Osteoclasts
Source: J Biol Chem. 2015 Dec 15;291(7):3439–54. doi: 10.1074/jbc.M115.677427 (PMC4751386; doi:10.1074/jbc.M115.677427)
Supplement: Supplemental Data [file supp_291_7_3439__index.html]

Involvement of receptor activator of nuclear factor kappa-B ligand-induced incomplete cytokinesis in polyploidization of osteoclasts — Involvement of Receptor Activator of Nuclear Factor-κB Ligand (RANKL)-induced Incomplete Cytokinesis in the Polyploidization of Osteoclasts — Osteoclast Polyploidization via Incomplete Cytokinesis — Supplemental Data 

# Involvement of Receptor Activator of Nuclear Factor-κB Ligand (RANKL)-induced Incomplete Cytokinesis in the Polyploidization of Osteoclasts

## Supplemental Data

- Supplemental legends (.pdf, 28 KB) - Legends for supplemental movies
- Supplemental movie 1 (.wmv, 1.8 MB) - Time-lapse imaging of a dTg-BMM that underwent incomplete cytokinesis after RANKL stimulation.
- Supplemental movie 2 (.wmv, 6.0 MB) - Time-lapse imaging of a dTg-BMM that went through cell fusion following incomplete cytokinesis.
- Supplemental movie 3 (.wmv, 928 KB) - Time-lapse imaging of dTg-BMMs that completed cytokinesis after incomplete cytokinesis.
- Supplemental movie 4 (.wmv, 7.0 MB) - Time-lapse imaging of a mononucleated polyploid dTg-BMM that went through cell fusion following incomplete cytokinesis.
- Supplemental movie 5 (.wmv, 4.4 MB) - FITC-labeled gelatin resorption assay
