# Supplementary material for: Involvement of Receptor Activator of Nuclear Factor-κB Ligand (RANKL)-induced Incomplete Cytokinesis in the Polyploidization of Osteoclasts
Source: J Biol Chem. 2015 Dec 15;291(7):3439–54. doi: 10.1074/jbc.M115.677427 (PMC4751386; doi:10.1074/jbc.M115.677427)
Supplement: Supplemental Data [file 10.1074_M115.677427_jbc.M115.677427-1.pdf]

Supplementary legends

Supplemental Movie 1

Time-lapse imaging of dTg-BMMs after RANKL stimulation. This imaging series was acquired by Deltavision microscope from an Olympus IX70 microscope equipped with a 20× objective. dTg-BMMs cultured in a glass-bottom dish were stimulated with RANKL (150 ng/ml) in the presence of M-CSF (60 ng/ml) and subjected to time-lapse imaging. A series of images was collected every 5 minutes at 37 °C. Yellow arrow indicates the cell that underwent incomplete cytokinesis described in Figure 4A.

Supplemental Movie 2

Time-lapse imaging of dTg-BMMs after RANKL stimulation. These imaging series were acquired by Deltavision microscope from an Olympus IX70 microscope equipped with a 20× objective. dTg-BMMs cultured in a glass-bottom dish were stimulated with RANKL (150 ng/ml) in the presence of M-CSF (60 ng/ml) and subjected to time-lapse imaging. A series of images was collected every 5 minutes at 37 °C. To acquire a large field of view, multipoint time-lapse imaging was performed. Shown is a connected field of view using After Effects (Adobe). Yellow arrow indicates the cell that underwent incomplete cytokinesis and subsequently underwent cell fusion described in Figure 5A.

Supplemental Movie 3

Time-lapse imaging of dTg-BMMs after RANKL stimulation. This imaging series was acquired by Deltavision microscope from an Olympus IX70 microscope equipped with a 20× objective. dTg-BMMs cultured in a glass-bottom dish were stimulated with RANKL (150 ng/ml) in the presence of M-CSF (60 ng/ml) and subjected to time-lapse imaging. A series of images was collected every 5 minutes at 37 °C. Yellow arrow indicates the cell that went through cytokinesis after incomplete cytokinesis described in Figure 4B.

Supplemental Movie 4

Time-lapse imaging of dTg-BMMs after RANKL stimulation. These imaging series were acquired by Deltavision microscope from an Olympus IX70 microscope equipped with a 20× objective. dTg-BMMs cultured in a glass-bottom dish were stimulated with RANKL (150 ng/ml) in the presence of M-CSF (60 ng/ml) and subjected to time-lapse imaging. A series of images was collected every 5 minutes at 37 °C. To acquire a large field of view, multipoint time-lapse imaging was performed. Shown is a connected field of view using After Effects (Adobe). Yellow arrow indicates the binucleated cell that completed cytokinesis and subsequently underwent cell fusion described in Figure 5B.

Supplemental Movie 5

FITC-labeled gelatin resorption assay. dTg-BMMs cultured in a FITC-labeled gelatin-coated glass-

bottom dish were stimulated with RANKL (150 ng/ml) and M-CSF (60 ng/ml) for 24 hours, then subjected to time-lapse imaging. A series of imagies was collected every 30 min at 37 °C. Left: fluorescence image. Right: Merged image. Yellow arrow indicates the mononucleated cell that underwent cell fusion, and Magenta arrow indicates the cell that underwent incomplete cytokinesis and re-enter S phase described in Figure 6E.
